# Usefulness of Running Wheel for Detection of Congestive Heart Failure in Dilated Cardiomyopathy Mouse Model

**DOI:** 10.1371/journal.pone.0055514

**Published:** 2013-01-31

**Authors:** Masami Sugihara, Fuminori Odagiri, Takeshi Suzuki, Takashi Murayama, Yuji Nakazato, Kana Unuma, Ken-ichi Yoshida, Hiroyuki Daida, Takashi Sakurai, Sachio Morimoto, Nagomi Kurebayashi

**Affiliations:** 1 Department of Cellular and Molecular Pharmacology, Juntendo University Graduate School of Medicine, Tokyo, Japan; 2 Department of Cardiovascular Medicine, Juntendo University Graduate School of Medicine, Tokyo, Japan; 3 Section of Forensic Medicine, Graduate School of Medical and Dental Sciences, Tokyo Medical and Dental University, Tokyo, Japan; 4 Department of Forensic Medicine, Graduate School of Medicine, The University of Tokyo, Tokyo, Japan; 5 Department of Clinical Pharmacology, Faculty of Medical Sciences, Kyushu University, Fukuoka, Japan; University of Tampere, Finland

## Abstract

**Background:**

Inherited dilated cardiomyopathy (DCM) is a progressive disease that often results in death from congestive heart failure (CHF) or sudden cardiac death (SCD). Mouse models with human DCM mutation are useful to investigate the developmental mechanisms of CHF and SCD, but knowledge of the severity of CHF in live mice is necessary. We aimed to diagnose CHF in live DCM model mice by measuring voluntary exercise using a running wheel and to determine causes of death in these mice.

**Methodology/Principal Findings:**

A knock-in mouse with a mutation in cardiac troponin T (ΔK210) (DCM mouse), which results in frequent death with a t_1/2_ of 70 to 90 days, was used as a DCM model. Until 2 months of age, average wheel-running activity was similar between wild-type and DCM mice (approximately 7 km/day). At approximately 3 months, some DCM mice demonstrated low running activity (LO: <1 km/day) while others maintained high running activity (HI: >5 km/day). In the LO group, the lung weight/body weight ratio was much higher than that in the other groups, and the lungs were infiltrated with hemosiderin-loaded alveolar macrophages. Furthermore, echocardiography showed more severe ventricular dilation and a lower ejection fraction, whereas Electrocardiography (ECG) revealed QRS widening. There were two patterns in the time courses of running activity before death in DCM mice: deaths with maintained activity and deaths with decreased activity.

**Conclusions/Significance:**

Our results indicate that DCM mice with low running activity developed severe CHF and that running wheels are useful for detection of CHF in mouse models. We found that approximately half of ΔK210 DCM mice die suddenly before onset of CHF, whereas others develop CHF, deteriorate within 10 to 20 days, and die.

## Introduction

Inherited dilated cardiomyopathy (DCM) is a progressive disease characterized by left ventricular dilatation and systolic dysfunction, and it is often associated with severe heart failure (HF) and sudden cardiac death (SCD) [1]–[3]. Although inherited DCM is reported to result primarily from mutations that cause weakness in force production [4], carriers of inherited DCM mutation do not always develop symptoms of HF at birth. Many are aware of symptoms of HF at some point in their life, which varies from young to old age, and the symptoms thereafter worsen [1], [3], [5]. Alternatively, some die suddenly before HF becomes evident [3]. Lethal arrhythmia is strongly suspected as a cause of sudden death: however, clear evidence for arrhythmia has not been documented in many cases. These reports raise important questions about how and when these symptoms appear in inherited DCM carriers. Because data in humans are confounded by various environmental and genetic factors, investigations with animal models of inherited DCM are required.

Genetically modified mouse models that have mutations for human inherited DCM were recently created [6], [7]. Among them, we have been investigating a knock-in mouse model with deletion mutation of K210 (ΔK210) in cardiac troponin T (*TNNT2*) [6], [8], which is identical to one of the human DCM mutations [4], [9]. Humans and mice with this mutation have lower Ca^2+^ sensitivity in force generation than do wild-type (WT) mice [4], [10]. Homozygous ΔK210 mice (DCM mice) have enlarged hearts and die with a t_1/2_ of 70–90 days after birth [6], [11], [12]. Telemetric ECG recordings showed torsades de pointes and ventricular fibrillation at the time of death [6], suggesting that arrhythmogenic changes occur. This model thus appears to resemble the phenotypes of human DCM [6], [11], [12] and is considered to be a good model for investigation of the pathophysiology of and therapeutic methods for DCM. Furthermore, we have found multiple types of ion channel remodeling in the hearts of these mice [8]; these channels may also be therapeutic targets in DCM. To understand the relationships among HF, arrhythmia, and various types of channel remodeling in inherited DCM, it is necessary to know the status of HF in individual live animals.

In terms of classification of HF, chronic HF can be divided into “compensated HF” and “decompensated HF” [13], [14]. In compensated HF, many overt features of fluid retention and pulmonary edema are absent. Decompensated HF refers to a deterioration, which may present either as an episode of pulmonary edema or as malaise, reduction in exercise tolerance, and increasing breathlessness on exertion [15]. The purpose of the present study was to detect the presence of congestive HF (CHF) or decompensated HF in an inherited DCM mouse model. One method for estimation of the severity of CHF in live animals is determination of exercise intolerance using a treadmill [16] or running wheel [17]–[19]. The treadmill exercise test, which involves forced exercise, is not suitable for DCM mice because they have a risk of SCD. For evaluation of CHF in live mice, we examined running wheel usage in this study and found a good correlation between voluntary exercise activity and severity of lung edema. On the contrary, other indicators of HF, such as atrial natriuretic peptide (ANP) and brain natriuretic peptide (BNP), were found to be more closely correlated with cardiac enlargement than CHF or lung edema. By measuring the time course of running activities in individual mice, we demonstrated that causes of death in ΔK210 DCM mice include 1) SCD without signs of CHF (≈50%) that can be attributed to ventricular arrhythmia, and 2) death related to severe CHF/decompensation (≈50%) that develops 10 to 20 days before death.

## Results

### Heart and lung weights in DCM mice

**Figure 1:**
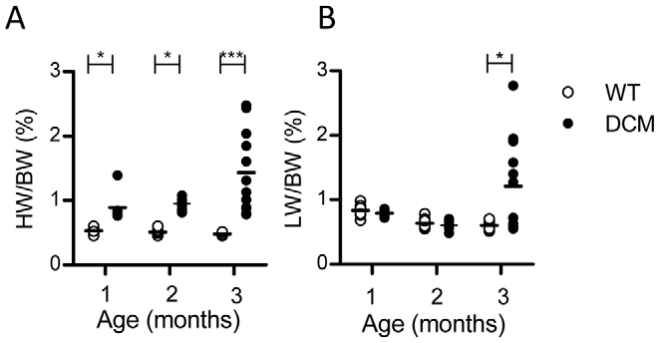
HW/BW and LW/BW ratios in individual WT and DCM mice at 1–3 months of age. ***A.*** HW/BW ratio. ***B.*** LW/BW ratio. Bars indicate means. *n* = 7–12. *P<0.05, ***P<0.001 vs. age-matched WT.

We first compared the heart to body weight (HW/BW) ratio with lung to body weight (LW/BW) ratio in WT and ΔK210 DCM mice at 1, 2, and 3 months of age (Fig. 1) before starting the experiments with the running wheel. The average HW/BW ratio was approximately 1.8-fold higher in DCM mice than in WT mice at 1 and 2 months of age (Fig. 1A, Table 1), and even at birth, as reported previously [6], [8]. The average HW/BW ratio at 3 months of age in DCM mice (1.44±0.63%) was 2.9-fold higher than that in WT mice (0.50±0.05%). Comparison of the 10–90 percentile ranges between 2- and 3-month-old DCM hearts indicates that the HW/BW ratio in the upper (90th percentile) range increased from 1.08% to 2.48%, while that in the lower (10th percentile) range remained constant at 0.81% to 0.78% (Fig. 1A, Table 1). These results suggest that further cardiac enlargement proceeded during the period between 2 and 3 months of age in some DCM mice.

**Table 1:** Averages and ranges of HW/BW and LW/BW ratios in individual WT and DCM mice at 1, 2, and 3 months of age.

Age	genotype	HW/BW (%) (10–90 percentile range)	LW/BW (%) (10–90 percentile range)
1 month	WT (n = 7)	0.53±0.05 (0.45–0.60)	0.83±0.10 (0.68–0.98)
	DCM (n = 7)	0.89±0.22* (0.76–1.39)	0.79±0.05 (0.72–0.86)
2 months	WT (n = 12)	0.51±0.03 (0.45–0.6)	0.63±0.08 (0.55–0.78)
	DCM (n = 12)	0.95±0.09* (0.81–1.08)	0.61±0.06 (0.49–0.70)
3 months	WT (n = 12)	0.50±0.05 (0.45–0.61)	0.60±0.08 (0.51–0.71)
	DCM (n = 12)	1.44±0.63*** (0.78–2.48)	1.21±0.73* (0.5–2.52)

Data are mean±SD with 10–90 percentile range in parenthesis.

* P<0.05,

*** P<0.001 vs. age-matched WT.

The LW/BW ratio was measured to detect signs of CHF (Fig. 1B, Table 1). There were no significant differences in LW/BW ratios between DCM and WT mice at 1 and 2 months of age (approximately 0.8±0.1% and 0.6±0.1%, respectively). At 3 months of age, however, the average LW/BW ratio was significantly higher in DCM (1.21±0.73%) than in WT (0.60±0.08%) mice. Close examination revealed that one half of the DCM mice showed LW/BW ratios similar to those of the WT mice (0.5–0.7%), whereas the remaining half of the DCM mice showed clearly higher LW/BW ratios (1–3%). This suggests that at 3 months of age, some DCM mice developed pulmonary edema, probably secondary to CHF.

### Voluntary wheel running activity in WT and DCM mice

**Figure 2:**
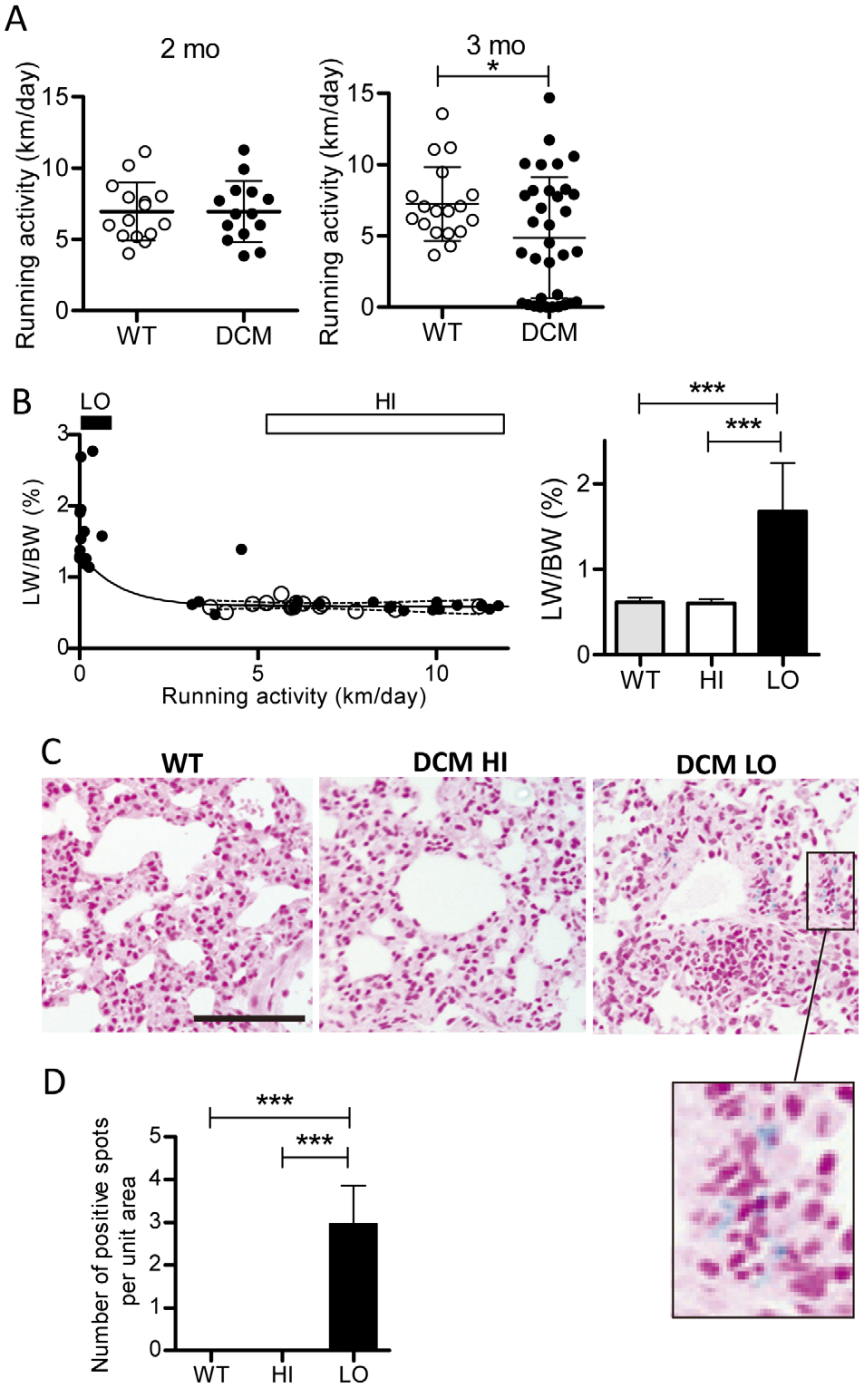
Relationship between running activity and LW/BW ratio and histological lung sections stained with Berlin blue. ***A*** **.** Running activity of WT and DCM mice at 2 (left) and 3 months of age (right). Bars are mean±SD. ***B*** **.** Relationship between running activity and LW/HW ratio in mice at approximately 3 months of age and average running activities in WT, DCM with high (>5 km/day, denoted “HI”) and low (<1 km/day, denoted “LO”) running activities (Left). Note that DCM mice (filled circles) with running activity of >5 km/day (HI) and all WT mice (open circles) showed similar LW/BW ratios, approximately 0.6% on average, whereas LW/BW ratios in all DCM mice with running activity of <1 km/day (LO) showed LW/BW ratios of >1%. ***C*** **.** Representative images of Berlin blue-stained lung sections from WT and HI and LO groups of DCM mice. Berlin blue-positive macrophages are detected only in sections from DCM mice with low activity. Bar indicates 100 µm. Inset: enlarged image. ***D.*** Average numbers of Berlin blue-positive alveolar macrophages per field of view of 0.2×0.25 mm^2^. Error bars are mean±SD (*n* = 4–5).

We hypothesized that development of CHF may be reflected by a decline in voluntary exercise activity. To test this hypothesis, DCM and WT mice at 5 weeks of age or older were housed in a cage with a running wheel for 48 consecutive hours every 8 to 10 days, and voluntary running distances were recorded. This standard exercise protocol did not affect the survival curve of DCM mice. After learning how to run on the running wheel during 1 to 2 trials (2–4 days), mice at 2 months of age showed almost constant running activity. This wheel running behavior was absolutely voluntary, not forced or reward-associated. The voluntary running distances were recorded until sacrifice at approximately 3 months of age. Until 2 months of age, running distances per day were similar between WT and DCM mice (mean±SD, 7±2 km/day; 10–90 percentile, 4–11 km/day) (Fig. 2A, left). Thereafter, at around 3 months of age, the running activity of WT mice remained unchanged (mean±SD, 7.2±2.6 km/day; 10–90 percentile, 4.2–11.4 km/day). On the other hand, the average running activity in DCM mice was significantly reduced; some DCM mice lost running activity, whereas others maintained a high activity (Fig. 2A right) (mean±SD, 4.9±4.2 km/day; 10–90 percentile, 0.02–10.3 km/day).

### Correlation between running activity and CHF

The relationship between running activity just before sacrifice and the LW/BW ratio was plotted (Fig. 2B left). Mice with lowered voluntary running activity showed larger LW/BW ratios; all mice with running activity of <1 km/day had considerably larger LW/BW ratios (mean±SD, 1.67±0.57%; range, 1.2–2.8%;), whereas all mice with running activity of >5 km/day had LW/BW ratios similar to those of the WT mice (mean±SD, 0.60±0.05; range, 0.5–0.7%) (Fig. 2B, left and right). A group of mice with intermittent running activities between 3 and 5 km/day included both normal and high LW/BW ratios. These results suggest that low running activity (<1 km/day) is a good indicator of CHF. To confirm this prediction and compare running activity with other indicators of CHF, we obtained data related to CHF in DCM mice with high (HI: >5 km/day) and low (LO: <1 km/day) activities in subsequent experiments.

In the next experiment, we examined whether hemosiderin deposition was present in histological lung sections in the LO group of DCM mice. Hemosiderin-loaded macrophages are thought to appear in the lung as a result of phagocytosis of red blood cells that have leaked from alveolar capillaries secondary to pressure overload in left ventricular HF [20]. Indeed, Berlin blue-positive alveolar macrophages were detected near capillaries in lungs from LO DCM mice, but never in lungs from HI DCM or WT mice (Fig. 2C and D).

### Correlation between running activity and other markers for HF

Several factors and markers, such as the size of the ventricular chamber, ECG signals, expression of the β-myosin heavy chain (β-MHC) isoform, and ANP and BNP levels, have been used to assess the severity of HF. We examined correlations of these factors with voluntary exercise activity.

**Figure 3:**
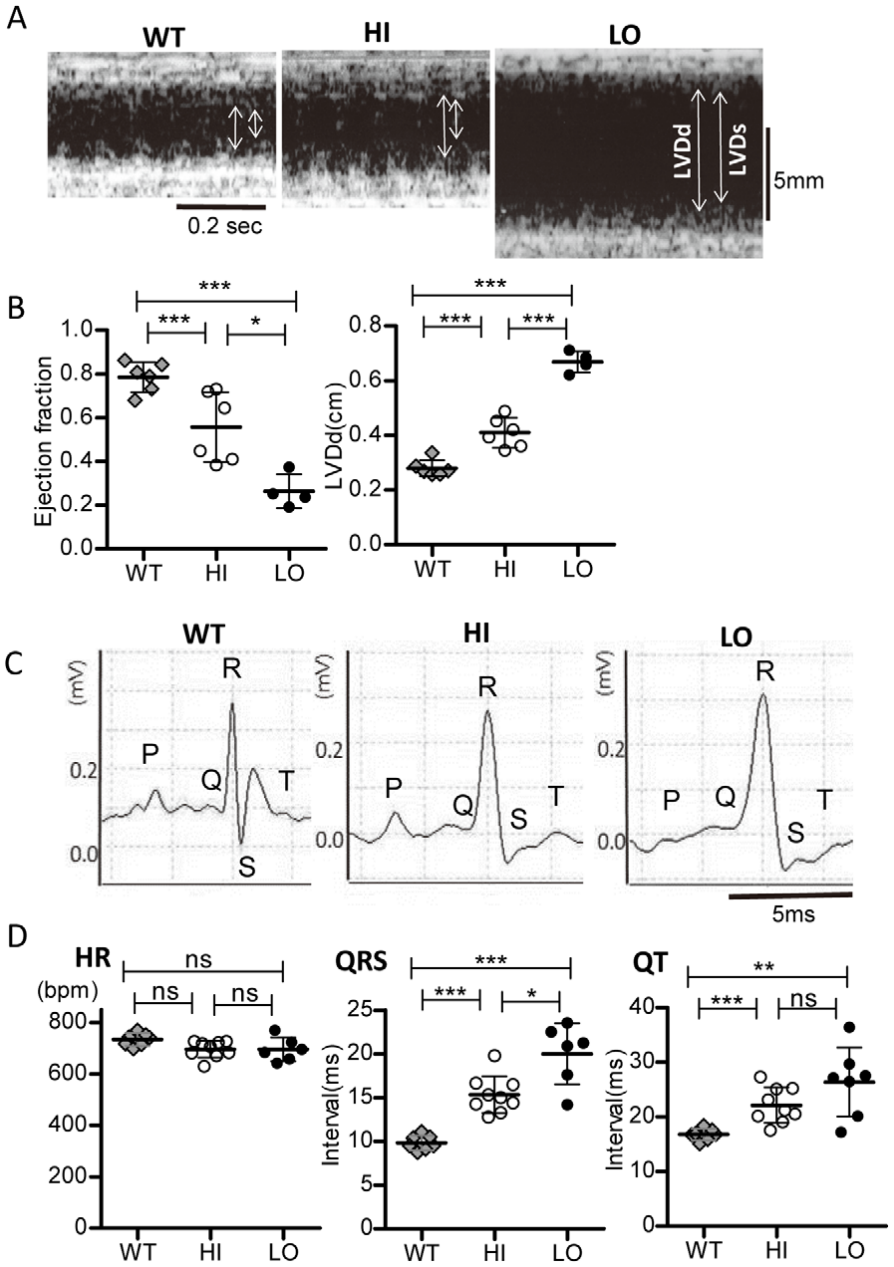
Echocardiography and ECG data from WT and DCM mice. ***A.*** Typical M-mode ultrasound cardiogram obtained from conscious WT and HI and LO DCM mice. ***B.*** Average LVDd (left) and EF (right). ***C*** **.** Typical ECG traces from WT and HI and LO DCM mice. ***D.*** Comparison of average heart rate (left), QRS (middle), and QT intervals (right) determined from conscious mice. Bars are mean±SD. *P<0.05, **P<0.01, ***P<0.001; ns: not significant.

First, echocardiography data were compared between the HI (*n* = 5) and LO (*n* = 4) groups (Fig. 3A and B). Because DCM mice are highly sensitive to anesthesia including inhalation and intravenous anesthetics, these data were obtained from conscious mice. Four of eight LO mice died before measurements were obtained, which was probably related to restraint [21]. A significantly higher left ventricular end-diastolic dimension (LVDd) and significantly lower ejection fraction (EF) were recorded in the LO mice that survived restraint than in the HI mice (Fig. 3B). Thus, the LO group showed decompensated HF with ventricular dilation, consistent with one of the major phenotypes of DCM. However, these results indicate that echocardiography increases the risk of death in severe HF model mice.

ECG data of WT, HI, and LO mice were also compared (Fig. 3C and D). No significant differences were observed in heart rates between WT and DCM mice (Fig. 3D, left). The average QRS interval was significantly longer in the LO group than in the WT and HI groups (Fig. 3D, middle), which is consistent with ventricular enlargement (Fig. 3B). The average QT interval was significantly longer in HI and LO DCM mice than in WT mice (Fig. 3D, right). Although the average QT interval was longer in LO than in HI mice, a significant difference was not detected (Fig. 3D, right).

**Figure 4:**
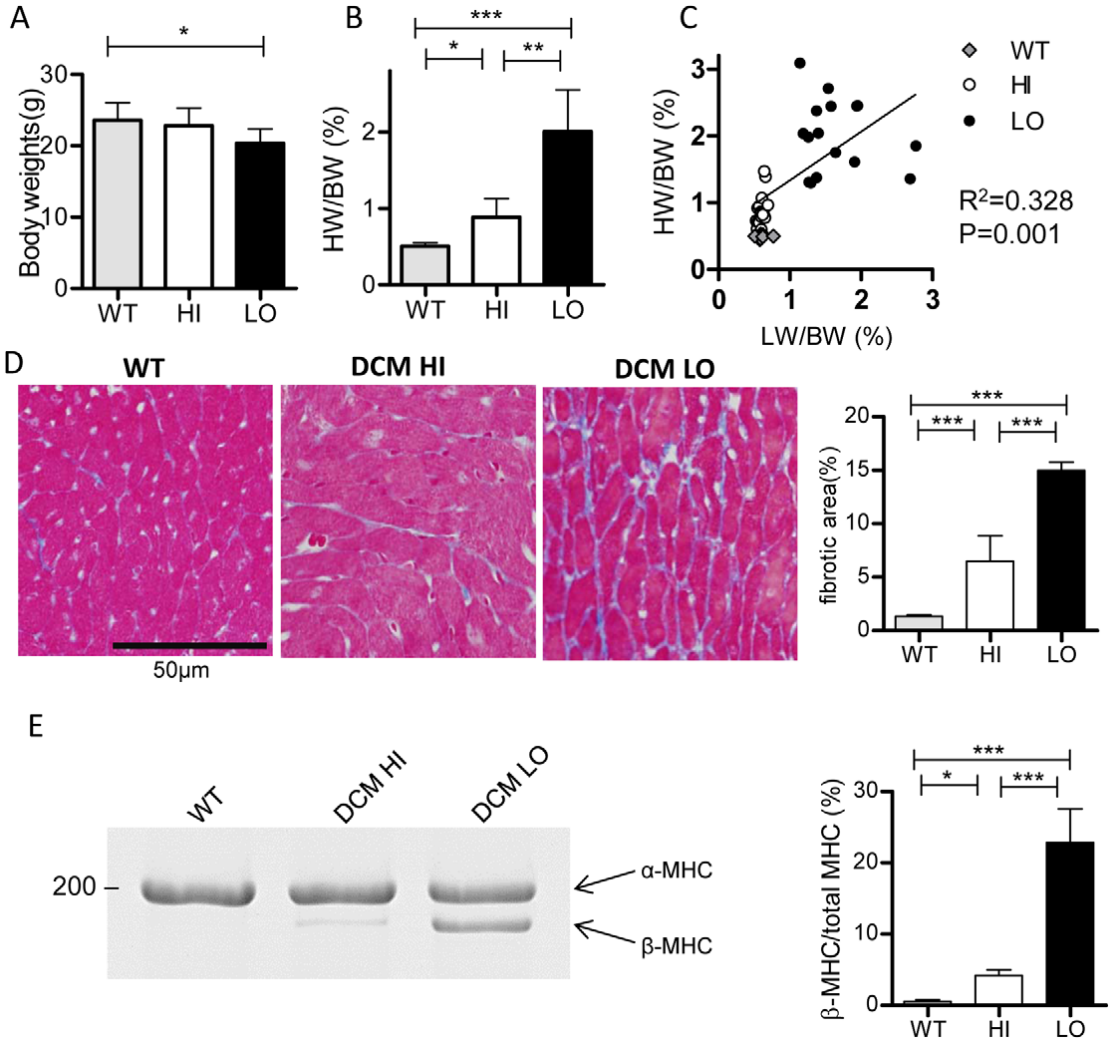
Heart weights, myocardial fibrosis, and protein expression level of β-MHC in myocardium. ***A.*** Body weight. ***B.*** HW/BW ratio. Data are mean±SD. *P<0.05, **P<0.01, ***P<0.001. ***C.*** HW/BW to LW/BW ratio relationship. Linear regression revealed a positive correlation between HW/BW and LW/BW in all DCM mice (P = 0.001). ***D.*** Histological analysis of the fibrosis in left ventricular myocardium. Connective tissues were stained blue with azan. Left three panels: representative data from WT and DCM HI and LO group mice. Right: Averaged fibrosis area. Data represent the mean±SD for three mice. ***E.*** Protein expression level of β-MHC in left ventricular myocardium. Left: Representative Coomassie brilliant blue staining pattern of α- and β-MHC in individual experiments. Right: Averaged expression levels. The expression level for β-MHC was normalized to the total MHC (α-MHC+β-MHC). Data are means±SD (WT: *n* = 4, DCM: *n* = 5). *P<0.05, **P<0.01, ***P<0.001 vs. WT.

We next compared body and heart weights between the HI and LO groups to directly examine whether cardiac enlargement was associated with decompensated HF in these DCM model mice. Body weights were similar among the three groups, although those of the LO group were slightly but significantly smaller than those of the WT group (Fig. 4A). The HW/BW ratio was significantly higher in the LO group than in the HI group; i.e., LO mice displayed more severe cardiac enlargement (Fig. 4B). Figure 4C shows the relationship between HW/BW and LW/BW ratios. There was a positive correlation between HW/BW and LW/BW ratios (r^2^ = 0.33) in DCM mice, suggesting that CHF and secondary cardiac dilation proceeded almost concurrently.

Cardiac fibrosis and the protein expression level of β-MHC were compared in the myocardium of the LO and HI groups because these factors were noticed in this DCM model as well as in other HF models [22], [23]. The LO group showed more advanced fibrosis than did the HI group (Fig. 4D), consistent with prolonged a QRS on the ECG. The protein expression level of β-MHC, expressed as the relative expression of β-MHC to total (α-MHC plus β-MHC) MHC, was markedly higher in the LO than in the HI group (Fig. 4E). These results confirm that cardiac fibrosis and up-regulation of β-MHC were much more severely pronounced in the deteriorated LO DCM mice.

**Figure 5:**
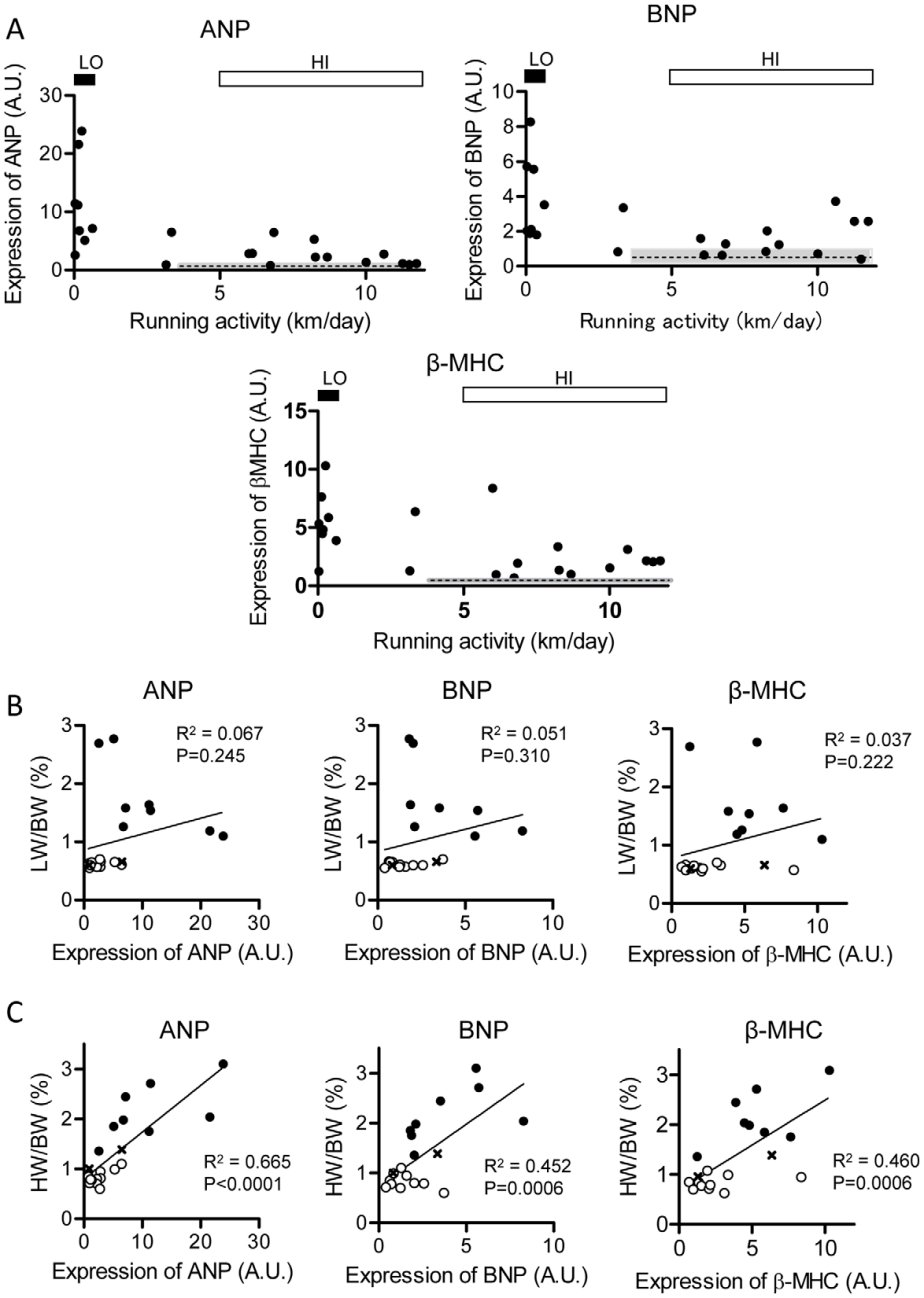
mRNA expression levels of ANP, BNP, and β-MHC in left myocardium. ***A.*** Relationship between mRNA expression levels and running activities in DCM mice. Upper left: ANP; upper right: BNP; bottom: β-MHC. Dotted line with gray bar is mean±SD of expression in WT myocardium. ***B.*** Relation of LW/BW ratio to expression of ANP (left), BNP (middle), and β-MHC (right) in DCM mice. ***C*** **.** Relation of HW/BW ratio to ANP (left), BNP (middle), and β-MHC levels (right). All data in ***A–C*** were obtained from the same set of DCM mice. In ***B*** and ***C***, HI: open circles (*n* = 12); LO: filled circles (*n* = 8); intermittent: crosses (*n* = 2). Lines indicate the least-squares fit for all DCM mice including HI, LO, and intermittent.

Gene expression levels of ANP, BNP, and β-MHC, which have been related to HF, were quantitatively determined in the same set of left ventricular myocardial preparations from the HI and LO groups. The expression level of ANP was higher in most of the HI mice than in the WT mice, and was further elevated in the LO mice (Fig. 5A, Table 2). Although a significant difference was detected between the HI and LO groups (Table 2), there was considerable overlap in individual values between the two groups (Fig. 5A). Similar results were obtained in terms of the expression levels of BNP and β-MHC (Fig. 5A, middle and right, respectively).

**Table 2:** Expression of ANP, BNP, and β-MHC levels and HW/BW and LW/BW ratios in myocardium from WT and HI and LO groups of DCM mice.

	ANP (A.U.)	BNP (A.U.)	β-MHC (A.U.)	HW/BW (%)	LW/BW (%)
WT (n = 11)	0.55±0.41	0.62±0.31	0.17±0.10	0.51±0.05	0.61±0.07
DCM HI (n = 12)	2.49±1.77	1.52±1.02	2.39±2.06*	0.82±0.14	0.62±0.04
DCM LO (n = 8)	11.20±7.71*** ^,^ †††	3.86±2.41*** ^,^ ††	5.45±2.68*** ^,^ ††	2.15±0.56*** ^,^ ††	1.72±0.65*** ^,^ †††

Data are mean±SD.

* P<0.05,

**P<0.01,

*** P<0.001 vs. WT,

†P<0.05 vs. HI,

††† P<0.01,

†† P<0.001 vs. HI. A.U.: Arbitrary unit.

To further evaluate quantitative correlations between expression levels and lung edema, the LW/BW ratio was plotted against the ANP level (Fig. 5B). Linear regression analysis revealed no significant correlation. Similar conclusions were obtained with the expression of BNP and β-MHC (Fig. 5B, middle and right). These results indicate that development of CHF/lung edema is not always reflected in the expression of ANP, BNP, and β-MHC.

On the contrary, there was a better quantitative correlation between the ANP level and cardiac enlargement than between the ANP level and lung edema; linear regression analysis revealed a significant correlation between the HW/BW ratio and ANP expression (Fig. 5C, left). There was also a significantly positive correlation between the HW/BW ratio and BNP level (Fig. 5C, middle) and between the HW/BW ratio and β-MHC level (right). These results indicate that the expression of ANP, BNP, and β-MHC better reflect cardiac enlargement than development of lung edema, which accompanies decompensated HF.

### Causes of death in DCM mice

**Figure 6:**
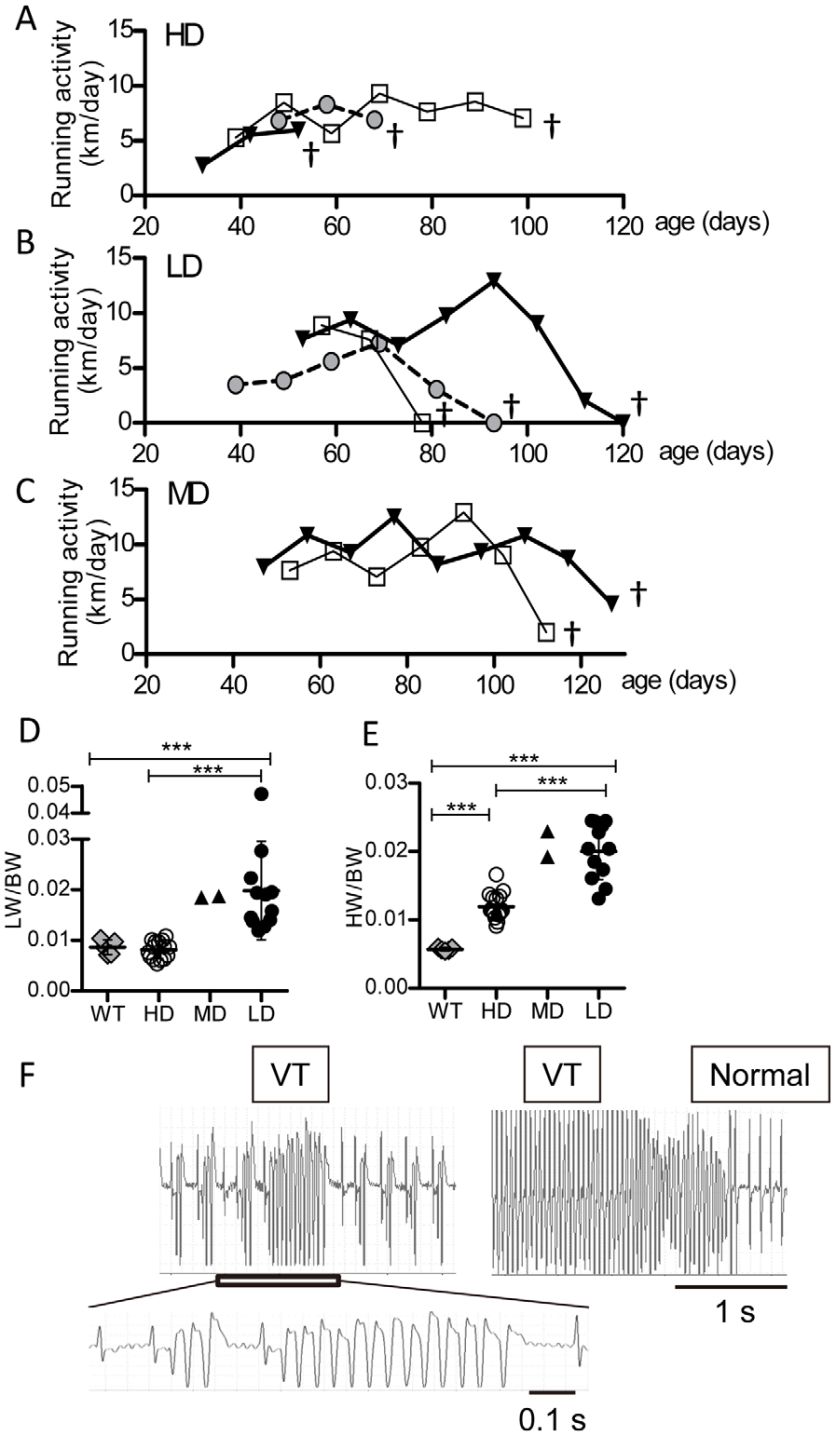
Time courses of voluntary running activity and causes of death in DCM mice. ***A.*** Typical time courses of running activity in three mice that maintained high activity (>5 km/day) and died suddenly. ***B.*** Instances of three mice that decreased running activity to <1 km/day and died. ***C.*** Instances of two mice that gradually lost running activity to between 1 and 5 km/day and died. Different symbols indicate different individual mice. ***D*** and ***E*** **.** LW/BW (*D*) and HW/BW (*E*) ratios in mice found dead in their cage. HD: mice with running activities of >5 km/day before death (*n* = 15). MD: mice that showed last running activity between 1 and 5 km (*n* = 2). LD: mice with activity of <1 km/day before death (*n* = 12). Bars are mean±SD. *P<0.05, **P<0.01, ***P<0.001. ***F.*** Typical ventricular arrhythmia recorded with telemetry ECG system from a 2.5-month-old mouse with high wheel running activity. Inset: ECG record with expanded time scale. Ventricular tachycardia (VT) repetitively occurred for 15 min in this mouse.

Finally, to determine whether ΔK210 DCM mice die with severe CHF or die suddenly without symptoms of CHF, we monitored the natural course of voluntary exercise activity in DCM mice until death. Figure 6A shows the representative data on running activity of DCM mice that died suddenly while maintaining high running activity. Among 29 mice examined, 15 maintained high running activity (>5 km/day) and died suddenly at 1.5 months or later. Their LW/BW ratios were similar to those of WT mice (see Fig. 6D; HD). In contrast, 12 DCM mice began to show decreased running activity at some point after 2 months, and then finally died within 10 to 20 days with running activity of <1 km/day (Fig. 6B). All of these mice showed high LW/BW ratios (see Fig. 6D; LD). To our surprise, the number of mice in the middle group (MD) (see Fig. 6C) between the HD and LD groups (1–5 km/day) was smaller than expected (only 2 of 29). They decreased their running activity to 20% to 40% of the basal activity and died with a high LW/BW ratio, indicating lung edema (Fig. 6C and D; MD). The fractions of SCD (HD) and CHF mice (LD+MD) estimated from running wheel activity were thus similar, approximately 50% each. The HW/BW ratio was smaller in the SCD group than in the CHF group (Fig. 6E) with a small overlap. Thus, low running activity not only reveals CHF, but also predicts the prognosis of DCM mice. The time course of running activity is also useful for the prediction.

A previous report showed torsades de pointes at the time of death in DCM mice [6]. In this study, similar ventricular arrhythmias were also captured at death in all DCM mice (*n* = 3) with telemetry ECG recordings. Furthermore, we recorded ventricular arrhythmia in mice that ran >5 km/day (*n* = 2). Figure 6F shows a representative ECG record obtained from a 2.5-month-old mouse with running activity of 7 km/day. This mouse survived ventricular arrhythmia that continued for 15 minutes and thereafter maintained high running activity for 1 month, occasionally showing less severe premature ventricular complexes and T wave alternans, and finally died of ventricular arrhythmia after development of CHF at 4 months. These results indicate that ventricular arrhythmia could occur in this DCM mouse model both before and after development of CHF, consistent with prolonged QT intervals before development of CHF. These results provide further evidence for lethal ventricular arrhythmia as a major cause of sudden death in inherited DCM before development of CHF.

## Discussion

A variety of mouse models of DCM have been created in recent years for the purposes of investigating the mechanisms of or therapy for DCM [5], [6], [24]–[26]. In this study, we confirmed that the ΔK210 knock-in mouse is one of the best models with which to study the cause of death, either CHF or SCD, in the natural course of inherited DCM. In the study of rodents as well as in clinical settings, it is critical to recognize the HF status and predict the prognosis of animals or patients. In this study, we demonstrated the usefulness of a running wheel for evaluation of CHF in live DCM model mice and examined the causes of death in these mice. Reduced activity was related to pulmonary congestion/edema, secondary ventricular dilation, and contractile dysfunction as confirmed by echocardiography, ECG, and postmortem examination.

### Comparison with other methods

Echocardiography is an established protocol for evaluation of cardiac function. We confirmed a close correlation among reduced running activity, ventricular dilation, and reduced contractile function (ejection fraction) as recorded by echocardiography. However, in basic studies of HF using mice, echocardiography may not always be applicable because it may frequently induce SCD, possibly owing to immobilization by physical restraint [21] or anesthesia.

ECG recording from the footpads of conscious mice is noninvasive. In this study, it revealed greater widening of the QRS interval in the LO group than in the HI group (Fig. 3), consistent with ventricular dilation (Fig. 4). However, the relatively large overlap of QRS values in the two groups indicates the limitation of ECG in the diagnosis and prediction of CHF. The same situation is also true for telemetric ECG recording, although it is a powerful tool with which to detect arrhythmias by continuous recording.

The animal treadmill system with an electrical shock stimulus has been widely used to estimate the severity of HF [16], [27], [28]. However, it may not be suitable for mouse models with a risk of SCD. In addition, there is high variation in treadmill running activities among mouse strains, and the C57BL/6 strain receives a significantly greater number of electrical shocks per minute compared with all other strains [29]. Among the procedures we utilized, we found that the wheel running test was the most effective and sensitive method for detection of CHF in mice. It is a low-cost, low-risk, noninvasive, easy, and animal-friendly method in experimental studies with mice. This method is dependent on the tendency toward voluntary exercise in mice [29]. We used the C57BL/6 strain, which comprise mice that run well on running wheels; however, most other breeds of mice can also be used for the voluntary running test [29]. This test is probably also applicable to other species that like running wheels, such as rats and hamsters. It is surprising that running on the wheel did not increase the death rate in DCM mice. This was probably because wheel running is not intense, but only a moderate daily exercise [29]. Thus, the reduction in running distance on the wheel is considered to reflect exercise intolerance.

It has been reported that the fetal gene program (ANP, BNP, and β-MHC) is re-activated in the hearts of DCM mice as well as those of mice in other HF models [30], indicating that cardiomyocytes undergo “pathological” hypertrophic remodeling in this DCM model [12]. Measurements of their expression levels revealed significant differences between the HI and LO groups; however, there was considerable overlap between them. Collectively, our results suggest that wheel running activity is a better indication of CHF than are ANP, BNP, and β-MHC levels in this model, whereas ANP, BNP, and β-MHC levels seem to be more closely related to cardiac enlargement/hypertrophy.

### Causes of death in the ΔK210 DCM mouse model and human inherited DCM patients

It has been shown that ΔK210 DCM mice frequently die after the age of 1.5 months [6], [8]. Ventricular arrhythmias were recorded at death in DCM mice with implanted telemetry ECG transmitters [6]. We consistently found that the ventricles of DCM mice revealed multiple types of ion channel remodeling at 2 months of age [8]. Our data in this study provide a detailed view of the causes of death in ΔK210 DCM model mice; sudden death with arrhythmias and death with decompensated HF occurred nearly equally. In DCM mice that developed CHF, the deterioration occurred 10 to 20 days before death.

The fractions of SCD in this DCM mouse model are consistent with those reported in populations of human patients with inherited DCM, in which causes of death are HF (approximately 50%) and SCD (approximately 40%) [2]. In humans, the cause of SCD in inherited DCM has been presumed to be lethal arrhythmia. However, uncertainties remain because in many cases, ECG recordings are not performed at the time of SCD, and lethal arrhythmia cannot be verified by autopsy. Our results strongly support the occurrence of lethal arrhythmia in human inherited DCM and suggest that some human DCM mutation carriers without diagnosis cannot recognize any abnormalities until lethal events occur. To prevent SCD, an early genetic diagnosis and start of therapy may be important for individuals with a family history. By applying the procedure developed in this study, we will be able to investigate therapeutic treatments to prevent SCD from compensated HF or rescue congestion/deterioration in decompensated HF separately and effectively.

### Study limitation and remaining problem

We were able to describe the time course of CHF development using a running wheel and predict death by CHF in DCM mice. However, it was difficult to predict SCD. In the mice with low running activity, the mechanisms underlying the rapid progression of decompensation were not elucidated, and thus await further study.

## Materials and Methods

### Animal model

All experiments were carried out in accordance with Ethics Committee guidelines and were approved by the Committee for Animal Experimentation of Juntendo University (approval number 230021). The investigation conformed to *Guiding Principles for the Care and Use of Animals in the Field of Physiological Sciences* (Physiological Society of Japan).

Knock-in mice with deletion mutation Lys-210 in their endogenous cardiac troponin T gene (*Tnnt2* ΔK210) were used as the DCM model animals [6]. These mice had been backcrossed with the C57BL/6J line for at least 10 generations and were maintained under specific pathogen-free conditions. Mixed-gender homozygous mutant and WT mice were obtained by crossing heterozygous mutant mice and were used as DCM and non-DCM models, respectively.

### Voluntary wheel running

Physical activity was evaluated by measuring voluntary wheel running activity. In a standard protocol, WT and DCM mice at 5 weeks of age or older were housed with free access to a running wheel (12-cm diameter, Mini Mitter, Bend, OR, USA) for 48 consecutive hours every 8 to 10 days, and voluntary running distances were recorded until sacrifice at approximately 3 months (77–90 days) of age. This standard exercise protocol did not affect the survival curve of DCM mice.

### Grouping of DCM mice according to running activities before sacrifice

To compare characteristics of body and heart data, DCM mice were divided into two groups according to their running activity just before sacrifice. DCM mice with running activity of >5 km per day just before sacrifice were placed into the high group (HI), and mice with declined activities of <1 km per day were placed into the low group (LO).

### ECG and echocardiography

ECG records of conscious mice were obtained before sacrifice of each animal with ECGenie (Mouse Specifics Inc., MA, USA), which noninvasively detected signals from the palmar and plantar aspects of the feet using footplate electrodes. Telemetric ECG recordings were carried out using an implantable telemetry system with ECG transmitters (ETA-F10) (Data Sciences International, USA), which was implanted in mice at 2 months of age. Using this telemetry system, we could obtain ECG records from mice that did not perform any wheel running exercise, but not, unfortunately, from running mice. Wheel running disturbs ECG recordings by contamination of skeletal muscle electrical activity (electromyogram). ECG records were analyzed with ECG Analysis software (ADInstruments, Japan). The LVDd and ejection fraction were also measured by transthoracic M-mode echocardiography (Sonos 5500; Philips Electronics Co., Amsterdam, The Netherlands) without anesthesia before mice were sacrificed.

### Body, heart, and lung weights

DCM and WT mice (1–3 months of age) were deeply anesthetized with pentobarbital sodium (100 mg/kg i.p.). The hearts and lungs were excised and rinsed in Krebs solution, and their weights were measured. The lung weight/body weight (LW/BW) and heart weight/body weight (HW/BW) ratios were calculated.

To determine causes of death, bodies of DCM mice found dead in their cages were weighed and autopsied within 0 to 24 hours after death. Their hearts and lungs were excised and weighed after clots were carefully removed. WT mice were euthanized by pentobarbital injection, and their bodies, hearts, and lungs were weighed 18 to 24 hours later.

### Lung and heart histology

Lungs were excised from 3-month-old WT and DCM mice, fixed in a 4% paraformaldehyde neutral-buffered solution, and embedded in paraffin. Sections at 6 µm were stained with Berlin blue. The number of Berlin blue-positive pulmonary alveolar macrophages per unit area was counted.

The hearts were excised and fixed by retrograde perfusion of a 4% paraformaldehyde neutral-buffered solution after perfusion with high-K^+^ Krebs solution (in mM: 120 NaCl, 25 KCl, 25 NaHCO_3_, 1 NaH_2_PO_4_, 2 CaCl_2_, 1 MgCl_2_, and 10 glucose, saturated with 95% O_2_–5% CO_2_). Fixed hearts were cut transversely at the midventricular level, embedded in paraffin, sectioned at 5 µm, and stained with azan. The extent of fibrosis in the left ventricular myocardium was quantified using the ImageJ program.

### Quantification of myosin heavy chain isoforms

Myosin heavy chain (MHC) isoforms in the left ventricular myocardium were separated on an sodium dodecyl sulfate polyacrylamide electrophoresis gel according to the method of Rundell et al. [31]. Protein bands were stained with Coomassie Brilliant Blue and acquired by LAS3000 (FujiFilm, Japan). Relative expression of the β-isoform (% of total MHC) was determined using MultiGauge software (FujiFilm, Japan).

### Gene expression of ANP, BNP, and β-MHC

Real-time PCR analysis was carried out as previously described [8]. Total RNA was isolated from the left ventricle using an RNeasy Fibrous Tissue Mini Kit (Qiagen) and treated with DNase I to prevent contamination with genomic DNA. First-strand cDNA was synthesized using a High-Capacity RNA-to-cDNA Kit (Applied Biosystems). The oligonucleotide primer sequences for ANP, BNP, and β-MHC were identical to those previously described [32]. The mRNA expression level of each gene in each sample was quantified relative to that of the glyceraldehyde 3-phosphate dehydrogenase (GAPDH) gene in the same sample.

### Statistics

Data are presented as means ± SD. Mean values were compared by one-way analysis of variance followed by a *post hoc* Kruskal-Wallis multiple comparison test for more than three groups. The difference between two mean values was analyzed with an unpaired Student's t-test. P values of <0.05 were considered significant. Other statistical parameters, such as the 10–90 percentile range, were obtained using GraphPad Prism software.
